# Functional Characterization of Novel *Lunatic Fringe* Variants in Spondylocostal Dysostosis Type-III with Scoliosis

**DOI:** 10.1155/2023/5989733

**Published:** 2023-07-11

**Authors:** Parker Wengryn, Karina da Costa Silveira, Connor Oborn, Carrie-Lynn Soltys, Alexander Beke, Inara Chacon-Fonseca, Nadirah Damseh, Marco Quesada Rodriguez, Ramses Badilla-Porras, Peter Kannu

**Affiliations:** ^1^Department of Medical Genetics, University of Alberta, 8-39 Medical Sciences Building 8614-114 Street, Edmonton, Alberta, Canada; ^2^Department of Medicine, University of Alberta, Edmonton, Canada; ^3^Department of Medical Genetics, University of Toronto, Toronto, Canada; ^4^Lakeridge Health Oshawa, Oshawa, Canada; ^5^Medical Genetics and Metabolics, Hospital Nacional de Niños, San José, Costa Rica

## Abstract

Scoliosis affects over four million Americans, with most cases having an idiopathic cause. Pathogenic variants in the *LUNATIC FRINGE* (*LFNG*) gene can cause spondylocostal dysostosis type-III (SCD3), which is a rare skeletal dysplasia characterized by the absence, fusion, or partial development of vertebrae and ribs. Acute restrictive lung disease and scoliosis may also be present in some cases. The variability in symptoms suggests that there may be other underlying pathological mechanisms that are yet to be discovered. We conducted an analysis of two novel *LFNG* variants, c.766G>A (p.G256S) and c.521G>A (p.R174H), that were observed in a patient with SCD3 phenotype and scoliosis. Characterizing these variants can help us better understand the relationship between genotype and phenotype. We assessed both variants for impaired glycosyltransferase activity, subcellular mislocalization, and aberrant pre-proprotein processing. Our results indicate that the p.G256S variant is enzymatically nonfunctional, while the p.R174H variant is functionally less effective. Both variants were correctly localized and processed. Our findings suggest that the hypomorphic variant (p.R174H) may have partially improved the patient's stature, as evidenced by a lower arm span-to-height ratio, increased height, and more vertebrae. However, this variant did not appear to have any effect on the severity of vertebral malformations, including scoliosis. Further research is necessary to determine the extent to which variations in LFNG activity affect the presentation of SCD3.

## 1. Introduction

Scoliosis, the pathological lateral curvature of the spine, can be a life-threatening condition that significantly impairs quality of life [1]. Complications include chronic pain, restrictive lung disease, and pulmonary hypertension. This cohort also experiences an increased prevalence of depression [2, 3], negative body image [4], and mood disorders. Although incidence varies by demography, 8% of Americans 40 or older presented with scoliosis as of 2011 [5]. Despite significant research investment, the etiology of scoliosis is largely unknown and termed idiopathic. Studying single-gene disorders associated with scoliosis can provide insights into genetic pathways which contribute to normal spinal development and thus are useful in identifying unexpected yet relevant factors which cause scoliosis.

Central to scoliosis pathobiology are somites. Somites are transient, embryonic spheres that rostrocaudally bud from the paraxial mesoderm in lateral pairs from days 20-35 during human development [6, 7]. These multipotent progenitor cells differentiate into the sclerotome, which gives rise to the vertebrae and ribs. Somitogenesis is characterized by the symmetrical, rostrocaudal elongation of the embryo during which “the clock and wavefront” mechanism acts [8–11]. The “wavefront” is an area of minimal fibroblast growth factor, wingless-and-Int-1, and retinoic acid signaling that permits the six-hour “clock” gene expression oscillations to differentiate precursor cells into the anterior and posterior somite [11–16]. As the embryo elongates posteriorly, it forces the midline posteriorly as well, allowing new progenitor cells to be acted upon. Somite symmetry is necessary for vertebral symmetry.

Clock oscillation is chiefly regulated by differential ligand binding to NOTCH1 during canonical NOTCH signaling [17–19]. LFNG, a Golgi-resident *β*-1,3-N-acetylglucosaminyltransferase, elongates fucose residues on epidermal growth factor- (EGF-) like repeats of the NOTCH1 extracellular domain (NECD). LFNG enhances NOTCH1 heterodimerization with delta-like ligand 1 (DLL1) [19, 20] causing pulses of NOTCH intracellular domain (NICD) endocytosis. The pulsatile nature of this process is critical for the expression of genes which encode the anterior somite [11–13, 15, 16]. In contrast, the genes encoding the posterior somite depend on sustained NICD endocytosis, which is mediated through the repression of *LFNG* by HES7 and increased heterodimerization of NOTCH1 with delta-like ligand 4 (DLL4) [21, 22]. Thus, LFNG plays a crucial role in controlling somite polarization and organization.

Spondylocostal dysostosis type-III (SCD3) is an autosomal recessive condition characterized by disorganized somites that result in short stature, missing or fused vertebrae, hemivertebrae, missing and fused ribs, scoliosis, and respiratory failure caused by adult-onset restrictive lung disease [23–27]. Biallelic pathogenic variants in *LFNG* have been reported in six probands in different reports [23–27] (RefSeq NM_001040167.2), three of which whose variants have been characterized *in vitro*. Of the six described probands, three were identified with homozygous variants, and the rest were compound heterozygotes. The c.564C>A (p.F188L) pathogenic *LFNG* variant was present in the homozygous state and led to protein mislocalization and impaired enzymatic function [26]. Perturbance of both function and localization decreased NOTCH activation and is suggested to have impaired oscillatory signaling during somitogenesis. Takeda et al. [27] described compound heterozygous *LFNG* variants (c.467T>G (p.L156R), c.856T>G (p.R286W)) which caused diminished glycosyltransferase activity. This lack of activity was also suggested to have caused aberrant somitogenesis. The final SCD3 case characterized at a molecular level described compound heterozygous *LFNG* variants (c.372delG (p.K124Nfs^∗^21), c.601G>A (p.D201N)) [24]. The frameshift variant was assumed to cause nonsense-mediated mRNA decay whereas the missense variant was found to diminish enzymatic function. Both variants would have prevented *LFNG* from regulating NOTCH signaling during somitogenesis. Both missense and frameshift *LFNG* variants caused SCD3 by inhibiting N-acetylglucosamine transfer to fucose on the NECD of NOTCH1.

Here, we describe the phenotype and characterization of two novel *LFNG* variants (c.766G>A (p.G256S), c.521G>A (p.R174H)) from a proband with an SCD3 phenotype. The variants are located *in trans* and have been classified as variants of uncertain significance (VUS). To support their role in causing the SCD3 phenotype, we present *in vitro* data assessing glycosyltransferase activity, protein localization, and pre-proprotein processing. Our analysis led to the identification of the first hypomorphic *LFNG* variant c.521G>A (p.R174H) associated with disease and characterization of the first compound heterozygote with both loss of function c.766G>A (p.G256S) and hypomorphic c.521G>A (p.R174H) variants. These findings provide new insights into the pathogenesis of SCD3 and highlight the importance of *LFNG* in somite development.

## 2. Methods

### 2.1. Exome Sequencing

Research-based exome sequencing was conducted with blood samples collected from the proband and parents. Sequencing was undertaken at the SickKids Hospital Center for Applied Genomics on the Illumina platform using the HiSeq4000 sequencer (100 bp PE, 6G/sample) and SureSelect V6 library prep kit (Agilent, USA) with 100 mean coverage. The reads were mapped to hg38 and genotypes were called using GATK 4. *Vcf* files were analyzed using the Franklin website (https://franklin.genoox.com/clinical-db/home). Filtering and prioritization of variants (indels, nonsense, missense, and splice variants) were conducted considering variants with frequencies lower than 1% in gnomAD (Genome Aggregation Database — http://gnomad.broadinstitute.org/) and EVS (Exome Variant Server—http://evs.gs.washington.edu/EVS/).

### 2.2. *In Silico* Variant Analysis

Variant pathogenicity was assessed under the ACMG classification system [28] with the web tools SIFT (Sorting Intolerant From Tolerant, https://sift.bii.a-star.edu.sg/), PolyPhen-2 (http://genetics.bwh.harvard.edu/pph2/), Align-GVGD (http://agvgd.hci.utah.edu/agvgd_input.php), CADD (Combined Annotation-Dependent Depletion, https://cadd.gs.washington.edu/info), and REVEL (Rare Exome Variant Ensemble Learner, https://sites.google.com/site/revelgenomics/).

### 2.3. *In Silico* Protein Modelling

The freely available online workflow ColabFold [29] was used to generate AlphaFold2 [30] derived structural models of LFNG wildtype, LFNG p.G256S, and LFNG p.R174H. One FASTA sequence for each variant was folded *ab initio* without any templates or relaxation steps. MMseqs2 was used for multiple sequence alignment. Five versions of each structure were folded and ranked by overall pLDDT scores; then, predicted finalized structures were visualized in the PyMOL Molecular Graphics System, version 2.0 (Schrödinger, LLC).

### 2.4. Cloning and Subcloning

Full-length *hLFNG* was amplified from *hLunatic Fringe VersaClone cDNA* (R&D, RDC1570; RefSeq NM_001040167.2 (clone NP_001035257)) through PCR (F: 5′-ATGCTCAAGCGTTGTGGACGAC-3, R: 5-GAAGATGGCAGTGCGGGGAC-3′), and identity was confirmed with Sanger sequencing (University of Alberta Molecular Biology Core). The amplicon was gel-extracted (Qiaex II Gel Extraction Kit, Qiagen, Cat: 20021) following manufacturer's protocol and subcloned into *pCR2.1-TOPO* (Thermo Fisher, K450002). Mach1 T1 phage-resistant chemically competent *E. coli* (Fisher Scientific, C86203) were used for transformation following the manufacturer's protocol without X-GAL due to LFNG toxicity. Plasmid DNA was extracted through miniprep (Wizard Plus SV Miniprep DNA Purification System, Promega, A1460) and then digested with Xho1 (Anza, Invitrogen) to allow for screening of insert directionality.

*pCR2.1-TOPO-hLFNG* and *p3XFLAG-myc-CMV24* (Sigma) were digested with HindIII (Anza, Invitrogen) and Xba1 (Anza, Invitrogen) and then gel-purified. The backbone of *p3XFLAG-myc-CMV24* and *hLFNG* (with short 5′ and 3′*pCR2.1-TOPO* sequences) was ligated with T4 DNA ligase (hereafter referred to as *LFNG-myc*) (Rapid DNA Ligation Kit, K1422) and then used to transform Mach1 *E. coli*. Plasmid DNA was extracted through miniprep and confirmed with Sanger sequencing.

*LFNG-myc* and *3XFLAG-hLFNG* (a kind gift from Dr. Shuji Mizumoto) [27] underwent site-directed mutagenesis to obtain the c.564C>A (F: 5′-CGTGGAGTATGACCGCTTAATCGAGTCCGGCA-3′, R: 5′-TGCCGGACTCGATTAAGCG GTCATACTCCACG-3′), c.766G>A (F: 5′-GTTTGCCACGGGCAGCGCTGGCTTCTG-3′, R: CAGAAGCCAGCGCTGCCCGTGGCAAAC-3′), and c.521G>A (F: 5′-CGCCCACAGCCACCAGGCGCTGT-3′, R: 5′-ACAGCGCCTGGT GGCTGTGGGCG-3) (QuikChange XL Site-Directed Mutagenesis Kit, Agilent, Cat: 200516-5) variants. We then transformed Mach1 *E. coli*, and plasmid DNA was extracted through miniprep and confirmed with Sanger sequencing.

Wildtype and variant *LFNG-myc* constructs were in-fusion cloned (In-Fusion Snap Assembly Master Mix with Competent Cells, Takara, 638952; a gift from Dr. Serhiy Havralov and Dr. Ordan Lehmann) into the *PCDNA3.1* backbone of *TRPML1-HA* [31] (gifted by Craig Montell, Addgene plasmid #18825; n2t.net/addgene:18825; RRID: Addgene 18825) as per manufacturer's instructions with Mach1 *E. coli* transformation. Plasmid DNA was extracted through miniprep and midiprep (ZymoPURE II Plasmid Midiprep Kit, Zymo, Cat: D4201) following manufacturers' protocols and confirmed with Sanger sequencing.

### 2.5. Cell Culture

HEK293T cells (ATCC, CRL-11268) were cultured in Dulbecco's Modified Eagle Medium (DMEM) (Gibco, 11995-065) with 10% fetal bovine serum (Sigma, F1051) and 1% Pen/Strep+glutamine (Gibco, 10378-016). NIH3T3 cells (ATCC, CRL-1658) were cultured in DMEM with 10% calf serum (ATCC, 302030) and 1% penicillin-streptomycin-glutamine (Gibco, 10378-016). Cells were regularly tested for mycoplasma contamination via PCR.

### 2.6. Western Blotting

2.5 × 10^5^ NIH3T3 cells were plated on 6-well plates for 24 hours and then transiently transfected with each of the *LFNG-HA* constructs using Lipofectamine LTX (Thermo Fisher, 15338100), following the manufacturer's instructions. After 48 hours, cells were scraped with 1% NP40 lysis buffer (20 mM Tris pH 7.4, 5 mM EDTA, 10 mM Na_4_P_2_O_7_, and 100 mM NaF), phosphatase inhibitor (Millipore, 524628), protease inhibitor (Sigma, P8340), and sodium orthovanadate. The cells were then sheared with a 26½ gauge needle and cleared by centrifugation at 1200 × g for 30 minutes. Each sample was quantified via BCA assay, and unadjusted protein samples were flash-frozen with liquid nitrogen or dry ice and placed at -80°C for long-term storage. Concentration-adjusted samples were run on an SDS polyacrylamide gel, transferred to a nitrocellulose membrane, and blocked in a 5% skim milk solution. Blots were rotated overnight in primary antibody at 4°C (mouse anti-FLAG-M2 (Sigma F1804), 1 : 1000; mouse anti-*β*-actin (Abcam AB6276, AC-15), 1 : 1000; and rabbit anti-HA (Cell Signaling C29F4), 1 : 1000). The next morning, blots were incubated in either HRP-linked goat anti-mouse IgG (Cell Signaling 7076S, 1 : 1000) or HRP-linked goat anti-rabbit IgG (Cell Signaling, 7074S) secondary antibody for one hour. Chemiluminescence was achieved through the Western Lightning ECL Plus kit (PerkinElmer) on a BioRad ChemiDoc. Densitometry was conducted in Image Lab 6.1 (BioRad) and exported to Microsoft Excel for aggregation.

### 2.7. Functional Assay

Functional analysis was conducted as previously reported with *3XFLAG-hLFNG* [27]. The protocol and plasmid were both generous gifts from Dr. Shuji Mizumoto, and the method description partly reproduces the wording. 2.0 × 10^6^ HEK293T cells were plated in poly-l-lysine-coated 10 cm dishes for 22 hours and then transiently transfected with each of the *3XFLAG-hLFNG* constructs using Lipofectamine 3000 (Thermo Fisher, L3000001). After 68 hours, the media were incubated with anti-FLAG M2 Affinity Gel (Sigma, A2220) for two hours at 4°C. 10 *μ*L of FLAG-bound resin was then incubated with 40 *μ*L of reaction mixture (50 mM 2-(N-morpholino) ethanesulfonic acid-NaOH (pH 6.5), 10 mM MnCl_2_, 0.1 mM UDP-GlcNAc (Promega, V7071), and 1 mM p-nitrophenyl-*α*-L-fucose (Sigma, N3628)) for 2 hours at 37°C. After returning to room temperature, 25 *μ*L of supernatant was mixed with 25 *μ*L of UDP Detection Mix as per manufacturer's instructions for one hour at room temperature. Luminescence was quantified with a TD-20/20 Luminometer (Turner Systems) in 1.5 mL Eppendorf tubes (Fisher Scientific) and compared to a standard curve of UDP concentration/luminescent intensity.

### 2.8. Immunofluorescence Microscopy

1 × 10^5^ NIH3T3 cells were seeded on glass coverslips coated with poly-l-lysine in 6-well plates for 24 hours. The next day, media was replaced, and cells were transfected with each of the *LFNG-HA* plasmid constructs using Lipofectamine LTX Reagent following the manufacturer's instructions. After 24 hours, media was removed, cells were fixed with 4% paraformaldehyde at room temperature for 15 minutes, and then permeabilized with 0.1% Triton-X100 for five minutes at room temperature. Samples were then blocked with 3% BSA for one hour, washed with PBS 3×, and incubated with rabbit anti-HA (Cell Signaling C29F4, 1 : 800) and mouse anti-GM130 (BD 610623, 1 : 800) primary antibodies for two hours. Cells were washed with PBS 3× and incubated with secondary antibodies for one hour (goat anti-mouse Alexa Fluor 594 (Thermo Fisher A11005, 1 : 500), goat anti-rabbit Alexa Fluor 488 (Thermo Fisher A11034, 1 : 500)). Cells were washed again with PBS 3×, stained with Hoechst 33342 at 1 *μ*g/mL (Molecular Probes, #H-3570) for 15 minutes, washed with PBS 3×, and mounted on slides with ProLong Gold Antifade Mountant (Invitrogen, P36930). Fluorescence microscopy was conducted at 60× in the spinning disk confocal microscope (Quorum Technologies) at the Cell Imaging Core at the University of Alberta. Images were processed and analyzed with Fiji software.

### 2.9. Statistical Analysis

To compare three or more groups at once, a one-way ANOVA was performed. Test assumptions were verified before test performance to maintain the internal validity of the results. Upon receiving a significant output from the one-way ANOVA (*p* < 0.05), the Bonferroni post hoc statistical analyses were conducted. Statistical tests were performed in Microsoft Excel and figures were created in GraphPad Prism version 8.0.0 for Windows. 

## 3. Results

### 3.1. Clinical History

**Figure 1:**
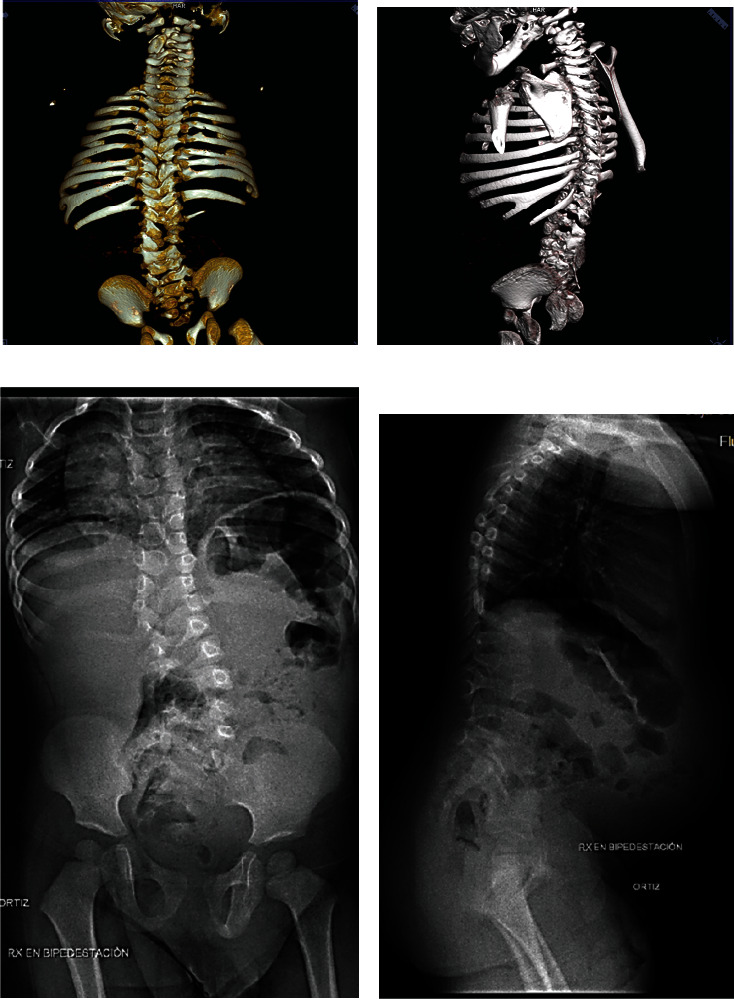
Computed tomography and X-ray imaging of the SCD3 proband. (a) Posterior CT image at three months. Note the disorganized vertebrae in the cervical, lumbar, and sacral spine and the right thoracic hemivertebrae. There are 10 left and 9 right asymmetrically set ribs with posterior proximal fusion and posterior distal fission. (b) 45-degree clockwise posterior CT image at three months. Note posterior rib fusion and fission as well as asymmetrical vertebral shape. (c) Posterior X-ray at one year and eight months. The posterior spine is characterized by the “pebble beach sign,” angulated vertebrae, and scoliosis. (d) Lateral X-ray at one year and eight months. Note the disorganized vertebral bodies in the thoracic and lumbar spine.

The proband was born by caesarian section after an uncomplicated pregnancy at 41 weeks of gestation. He did not require resuscitation and his APGAR scores were 9^1^ and 9^5^. His head circumference and weight were at or above the 50^th^ percentile, and his length fell between the 10^th^ to 25^th^ percentiles. He had a wide anterior and posterior fontanelle with a separated sagittal suture, abnormal spinal curvature with short neck, congenital torticollis, and a wide and short thorax. A thoracic spina bifida was present. Cardiovascular and respiratory examinations were normal. Investigations, including head and abdominal ultrasound, were also normal. Spinal computed tomography scans revealed large vertebral segmentation anomalies with rib malalignment and fusions (Figures 1(a) and 1(b)). At one year and eight months, spinal radiographs showed vertebral segmental defects and rib fusions (9 right ribs and 10 left) (Figures 1(c) and 1(d)). He had a left 38-degree scoliosis at L1/S1, 44-degree lordosis at L1/S1, and 32-degree kyphosis at T2/T12. On his last evaluation at two years and four months, his height was between the 10^th^ and 25^th^ percentiles, and the arm span-to-height ratio was 1.075. He had an asymmetric thorax with pectus carinatum but no major respiratory issues. There was no limitation of neck and limb movement, and he was developmentally appropriate.

His parents were a healthy (mother aged 35 years and father aged 39 years) and nonconsanguineous couple of Costa Rican background. His father had two healthy children with a previous partner. There was no family history of significance. Parental height was 195 cm and 150 cm for his father and mother, respectively.

### 3.2. Genetic and Proteomic Analysis of Novel *LFNG* Variants

Whole exome sequencing revealed two *LFNG* variants c.521G>A (p.R174H) and c.766G>A (p.G256S) (RefSeq NM_001040167.2). These two variants were novel and *in trans* (see Table 1). The first missense change, located in exon 3 of *LFNG*, was paternally inherited. This change replaced arginine with histidine at codon 174 of the LFNG protein (p.R174H) and was rare in population databases (7-2525258-G-A, gnomAD v3.1.2, 6.57 × 10^−6^). The second missense variant was maternally inherited and in exon 5. The glycine at codon 256 was replaced with serine (p.G256S). This variant was also rare in population databases (7-2525715-G-A, gnomAD v3.1.2, 6.57 × 10^−6^).

Both variants were analyzed in SIFT, PolyPhen-2, Align-GVGD, CADD, and REVEL to generate *in silico* estimations of possible protein disruption (Table 1). SIFT determines outcomes of substitutions by sequence homology and physicochemical properties where arbitrary outcome scores of <0.05 are predicted to be deleterious [32]. The p.R174H substitution was predicted to be tolerated with a score of 0.22, whereas the p.G256S substitution was predicted to be deleterious with a score of 0.00. Using PolyPhen-2, we assessed the effect of amino acid substitutions by analyzing sequence and structural homology as well as existing Pfam annotations. Scores are given from 0.0 to 1.0 as increasing probabilities that a variant is deleterious [33]. The p.R174H and p.G256S substitutions were given scores ranging from 0.746 to 0.970 and 1.00 to 1.00, respectively. Align-GVGD was then employed to assess the effect of each substitution in the context of proteomic sequence alignment and biophysical amino acid properties [34]. Scores are classed into one of seven categories from class C0 (benign) to class C65 (pathogenic). The p.R174H substitution was categorized as class C25 (GV 0, GD 28.2), whereas the p.G256S substitution was categorized as class C55 (GV 0, GD 55.3). CADD was utilized to determine the relative genome-wide deleteriousness of each variant [35]. Both p.R174H and p.G256S scored 25.6, indicating that they were within the top 0.4% of deleterious human genome variants. Finally, we used REVEL, a program which aggregates 13 variant prediction algorithms into one score which is scaled from 0.0 (benign) to 1.0 (pathogenic) [36]. p.R174H and p.G256S received scores of 0.515 and 0.889, respectively. Both c.521G>A (p.R174H) (PM2, PP2, and PP4) and c.766G>A (p.G256S) (PM2, PP2, PP3, and PP4) fell outside of ACMG variant classification guidelines and thus were classified as VUS [28].

**Figure 2:**
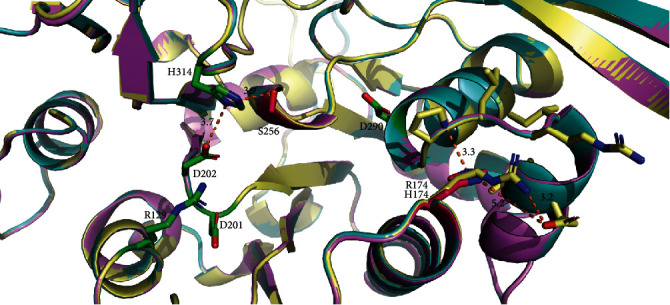
AlphaFold2 models of wildtype, p.R174, and p.G256S LFNG enzymes with highlighted residues. Wildtype residues are labeled yellow, p.R174 is labeled purple, and p.G256S is labeled turquoise. Important active site residues have been identified with a green color, p.H314 and p.D202 chelate Mn^2+^, p.R129 and p.D201 are responsible for substrate binding, and finally, D290 is the catalytic residue. Both substituted residues are highlighted with a bright pink color at 174 and 256. Position 174 demonstrates canonical arginine positioning overlaid with the histidine substitution. Measurements are taken in angstroms to show relative positioning in a 3D space and important nearby residues.

To qualitatively examine whether these variants cause structural perturbation *in silico*, we generated AlphaFold2-based structural models of WT LFNG, p.R174H, and p.G256S and aligned them in PyMOL2 (Figure 2). No structural changes were identified as both p.R174H and p.G256S LFNG substitutions maximally overlapped with WT LFNG.

### 3.3. The Novel *LFNG* Alleles Are Functionally Hypomorphic and Null

**Figure 3:**
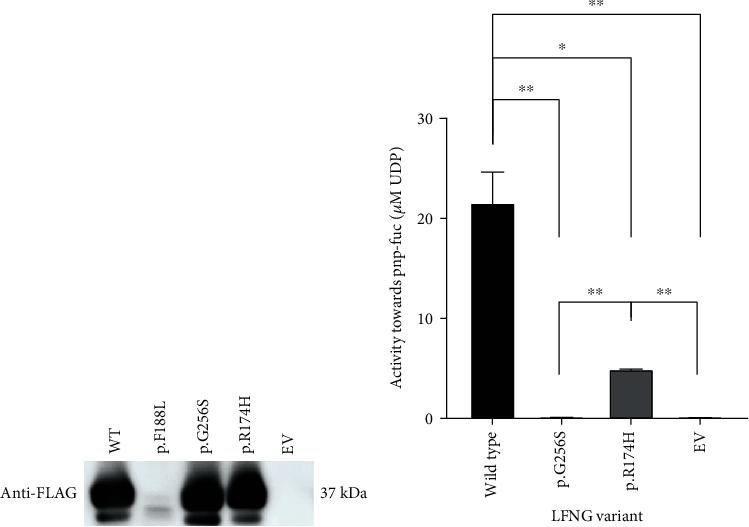
The p.G256S and p.R174H LFNG substitutions are null and hypomorphic, respectively. (a) Western blot of anti-FLAG immunoprecipitated 3XFLAG-LFNG protein. 3XFLAG-LFNG was purified with anti-FLAG agarose resin and disassociated with SDS buffer, and 20 *μ*L of each sample was run on a 10% SDS gel. Protein was detected with anti-FLAG primary antibody and HRP-linked anti-mouse IgG secondary. (b) GlcNAc transferase activity of LFNG variant protein isolated from media. One-way ANOVA indicated a significant difference between groups (*F* (3, 8) = 40.6, *p* = 3.5 × 10^−5^), and Bonferroni-adjusted (*α* = 8.3 × 10^−3^) post hoc analyses indicated that WT was significantly different from p.R174H, p.G256S, and E.V. p.R174H was significantly more intense than p.G256S and EV. There was no statistically significant difference between p.G256S and EV (*p* = 6.8 × 10^−1^). Values are means ± S.E of three independent experiments (*N* = 3) plated in triplicate. ^∗^*p* < 8.3 × 10^−3^ and ^∗∗^*p* < 2.5 × 10^−3^.

To investigate the effect of each *LFNG* variant *in vitro*, glycosyltransferase activity, protein processing, and intracellular localization were assessed. Previous work suggests that these factors contribute to SCD-III manifestation [24, 26, 27, 37, 38]. Glycosyltransferase activity was assessed using previously reported methods [27]. Briefly, HEK293T cells were transiently transfected with a shortened version of *LFNG* cDNA fused to a 3′ pre-protrypsin leader sequence (*3XFLAG-hLFNG*). Western blotting ensured similar secretion between conditions (Figure 3(a)). The WT, p.G256S, and p.R174H conditions appeared similar in band intensity whereas the functionally inactive variant p.F188L [26] was not secreted into media. We used the empty vector (EV) backbone as the negative control in line with the work of Takeda et al. [27].

The glycosyltransferase reaction was undertaken by incubating 3XFLAG-LFNG bound anti-FLAG agarose resin with a reaction mixture containing UDP-GlcNAc (donor) and p-nitrophenyl-*α*-L-fucose (acceptor). Here, one UDP molecule was released for each GlcNAc transfer, and this UDP was quantified via luminescence post-reaction. Quantitative analyses indicated that WT luminescence was significantly more intense than p.R174H (*p* = 6.5 × 10^−3^), p.G256S (*p* = 2.5 × 10^−3^), and E.V. (*p* = 2.5 × 10^−3^) (Figure 3(b)). The data also indicated that p.R174H luminescence was significantly more intense than both p.G256S (*p* = 7.5 × 10^−6^) and E.V. (*p* = 7.5 × 10^−6^). Ultimately, the data indicated that p.R174H GlcNAc transferase activity lied between WT and inactive levels and it was the first observation of a patient-derived LFNG substitution causing hypomorphic function.

### 3.4. Protein Processing Appears Normal with p.G256S and p.R174H LFNG

With the identification of a partially active *LFNG* variant, it was necessary to rule out that aberrant pre-proprocessing or protein mislocalization could cause the phenotype. LFNG contains an N-terminal type-II transmembrane domain that is cleaved at (K/R)XX(K/R) sites by subtilisin-like proprotein convertase family (SPC) proteins, specifically SPC6 [37, 38]. LFNG possesses two cleavable sites within its 86-residue transmembrane domain, RGRR (37 to 40) and RARR (83 to 86). Pre-proprocessing leads to LFNG exocytosis and allows for fine-tuning of clock cycle oscillations [37, 38]. Concordantly, these works suggest that aberrant cleavage impairs somitogenesis and causes SCD3 phenotype.

**Figure 4:**
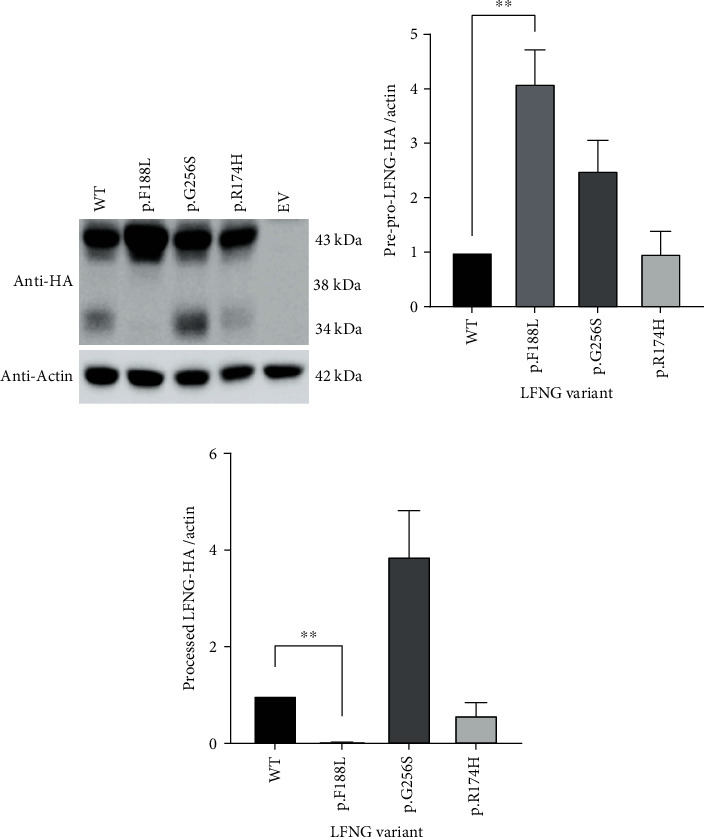
The p.G256S and p.R174H LFNG substitutions do not lead to aberrant protein processing. (a) Western blot of cell lysate from *LFNG-HA* transiently transfected NIH3T3 cells. Cell lysates were run on a 10% SDS gel, and membranes were incubated with anti-HA primary and anti-rabbit IgG secondary antibodies, or anti-actin primary and anti-mouse IgG secondary antibodies. (b, c) Densitometry analysis of pre-pro-LFNG-HA (b) or processed (c) LFNG-HA bands. Band intensities were normalized to actin. One-way ANOVA indicated a statistically significant difference between all conditions in both pre-pro-LFNG-HA (*F* (3, 8) = 9.1, *p* = 5.9 × 10^−3^) and processed LFNG-HA (*F* (3, 8) = 21.2, *p* = 4.4 × 10^−5^). Bonferroni-adjusted (*α* = 1.3 × 10^−2^) post hoc analyses indicated that the pre-pro-LFNG-HA p.F188L bands were significantly more intense than WT, and the processed LFNG-HA p.F188L bands were significantly less intense than WT. No other conditions were significantly different from WT. Values are means ± S.E of three independent experiments (*N* = 3) plated in triplicate. ^∗∗^*p* < 1.3 × 10^−2^.

To qualitatively and semiquantitatively assess each variant's effect on protein processing, intracellular protein lysate from NIH3T3 cells transiently transfected with 3′*HA-*fused *LFNG* expression plasmids (*LFNG-HA*) was western-blotted. The predicted molecular weights of LFNG-HA were 42.8 kDa (pre-pro-LFNG), 38.4 kDa (pro-LFNG), and 34.1 kDa (processed LFNG). The previously characterized p.F188L LFNG substitution [26] was also assessed to test whether protein processing and localization were linked. Qualitatively, both p.F188L and p.G256S exhibited increased pre-proprotein band intensity compared to WT (Figure 4(a)). However, densitometric analyses indicated that only p.F188L was significantly different from WT (*p* = 9.0 × 10^−3^) (Figure 4(b)). There were no bands in the preprocessed 38.4 kDa range for any of the conditions (Figure 4(a)). Finally, WT, p.G256S, and p.R174H conditions had faint bands at the processed protein weight of approximately 34.1 kDa (Figure 4(a)). Qualitatively, the p.F188L condition lacked this signal whereas in the p.G256S condition, it was more intense (Figure 4(a)). Statistical analysis indicated a significant difference in band intensity between WT and p.F188L (*p* = 9.0 × 10^−9^) (Figure 4(c)).

### 3.5. The p.G256S and p.R147H LFNG Substitutions Do Not Affect Subcellular Localization

**Figure 5:**
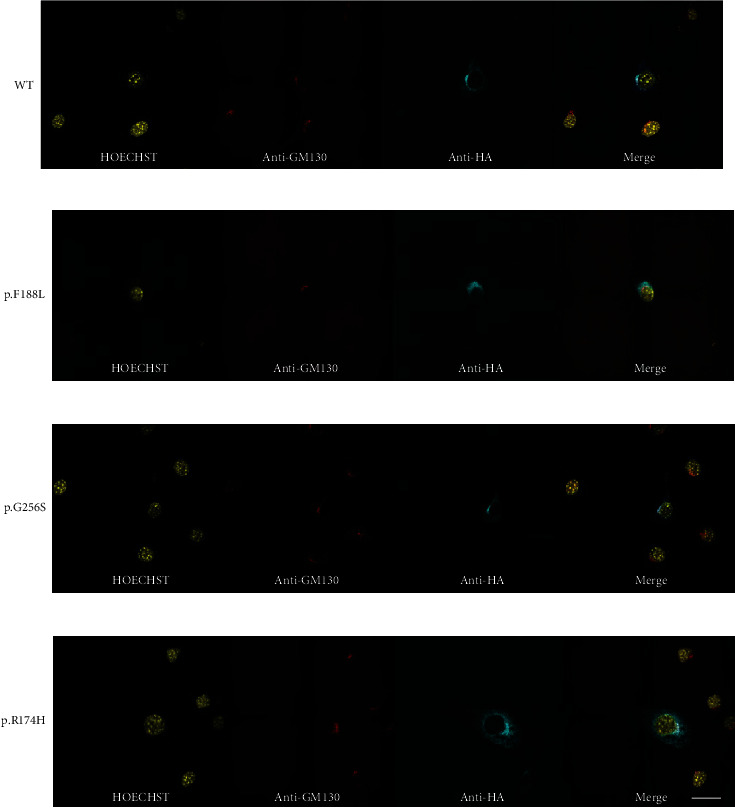
The p.G256S and p.R174H LFNG substitutions localize to the Golgi. (a) WT, p.F188L (b), p.G256S (c), and p.R174H (d) *LFNG-HA* variants transiently transfected NIH3T3 cells. Cells were incubated with Hoechst, rabbit anti-HA, and mouse anti-GM130 primary antibodies and then again with fluorescent secondary antibodies. The fluorescent signal was artificially altered to be color-blind friendly. Note the absence of signal colocalization in (b). Images are representative samples of three independent experiments (*N* = 3). Scale bar, 30 *μ*m.

Finally, we asked whether the variants could cause mislocalization of LFNG [26, 39]. NIH3T3 cells were transiently transfected with the *LFNG-HA* constructs. p.F188L was employed as a negative control as it is the only substitution previously shown to cause LFNG mislocalization [26]. Qualitative analysis indicated anti-HA and anti-GM130 signal colocalization in the WT, p.G256S, and p.R174H conditions (Figures 5(a), 5(c), and 5(d)). The anti-HA and anti-GM130 signals did not colocalize in the p.F188L condition, supporting previous results (Figure 5(b)) [26].

## 4. Discussion

*LFNG* is of interest due to its critical role in somitogenesis and the pronounced, yet varied presentation caused by pathogenic variants. Here, we identified a proband with SCD3 phenotype and two *LFNG* VUS according to the ACMG classification system. Since SCD3 is an autosomal recessive disorder, the recurrence risk is 25%. However, VUS cannot be used for clinical decision-making to inform reproductive risk management. Our work, therefore, is aimed at determining whether the p.G256S and p.R174H LFNG substitutions were pathogenic. Our functional data indicate that the p.G256S and p.R174H substitutions are null and hypomorphic, respectively. This data acts as PS3 (functional) ACMG evidence for each variant, elevating their status to likely pathogenic [28]. Therefore, our data support the hypothesis that these *LFNG* variants cause the SCD3 phenotype enabling the parents to access prenatal genetic testing or preimplantation genetic diagnosis in a subsequent pregnancy.

### 4.1. *In Silico* Mechanisms of Functional Perturbance

The biochemical mechanism of p.R174H hypomorphism appears to be reminiscent of a manic fringe- (MFNG-) like substitution. Specifically, the PolyPhen-2 multiple sequence alignment of fringe enzymes showed that p.R174H substitutes a lunatic-specific conserved residue (arginine) for a manic-specific conserved residue (histidine) (Supplementary Figure 1). MFNG is significantly less active than LFNG [39], and the functional data support that p.R174H follows this trend (Figure 3(b)). *In vitro*, it would be interesting to test (1) if histidine interacts with similar residues in both cases, (2) if there are similar structural features of both enzymes, and (3) if these interactions are the cause of lowered activity. These tests could aid in identifying amino acid changes which potentiate the increased activity of LFNG relative to MFNG and RFNG. From an evolutionary perspective, this may begin to elucidate the varied developmental roles of fringe family proteins across species.

Structurally, the core of each AlphaFold2 model has maximal overlap with WT (Figure 2). However, these models only account for visual structural changes, not functional ones. For example, although the p.R174H substitution is conservative, it may cause changes in hydrogen bonding due to the Golgi's slightly acidic pH (6.0 to 6.8) [40]. This is because histidine is deprotonated within this pH range whereas arginine is protonated. Such an effect may account for both maximal structural overlap and varied results across *in silico* functional analyses (Table 1). Contrastingly, although p.G256S is a nonconservative substitution, its location may allow for a WT-like structure (Figure 2) since residue 256 exists in a small bend to the opening of LFNG's active site. When glycine is substituted with serine, the new hydroxyl group may interact with the adjacent p.H314 which facilitates Mn^2+^ cofactor coordination (Figure 2). The distance between p.H314 and its native binding partner p.D202 is similar to the distance between p.H314 and p.S256 (3.7 Å and 3.9 Å, respectively). Therefore, serine's highly polar hydroxyl group could destabilize p.H314 Mn^2+^ coordination or even sequester it on the “wrong” side of this residue. This hypothesis would account for structural similarities (Figure 2) and damaging effects of the substitution (Table 1 and Figure 3(b)).

### 4.2. Mislocalization Prevents Protein Processing

We were unable to attribute mislocalization or aberrant pre-proprocessing to disease in this study. Both p.G256S and p.R174H were normally pre-proprocessed and present in the Golgi (Figures 4 and 5). Interestingly, however, the negative control p.F188L was aberrantly processed and mislocalized (Figures 4 and 5). Previous work only indicated that this variant mislocalizes [26]. Here, we propose that the p.F188L variant is not pre-proprocessed nor secreted because it is mislocalized. Primarily, there is an intracellular accumulation of pre-pro-LFNG-HA (Figures 4(a) and 4(b)) and an absence of processed LFNG-HA (Figures 4(a) and 4(c)). Furthermore, the p.F188L protein did not colocalize with the Golgi (Figure 5(b)). This suggests that the variant protein was not translocated to the Golgi and therefore was not cleaved by the Golgi-resident SPC6. Crucially, it is unlikely that processed LFNG p.F188L is absent due to secretion because 3XFLAG-hLFNG p.F188L was absent from media in our work (Figure 3(a)) and elsewhere [26, 27]. The combination of evidence suggests that p.F188L inhibits the ability of LFNG to translocate to the Golgi thus preventing its processing and secretion. It was previously hypothesized that this residue may play a role in ER export and Golgi transport [26], and current data supports this hypothesis. Future work should investigate molecular mechanisms behind the unknown role of this region. Pre-proprocessing is a key modifier of clock timing during somitogenesis, and it has been strongly suggested that the perturbance could lead to malformed somites [37, 38]. Therefore, it will also be important to test whether the mislocalization of otherwise functional LFNG causes SCD3 phenotypes in humans.

### 4.3. Partial Rescue of the Proband's Phenotype

Our evidence suggests that impaired glycosyltransferase activity alone is responsible for the proband's phenotype. Importantly, the proband in this study has two *LFNG* variants *in trans*; one of these variants is hypomorphic (p.R174H) and the other is null (p.G256S). It is interesting to consider whether partial activity from the hypomorphic variant may have modestly rescued the proband's phenotype. Such an effect has been documented in murine models of SCD3 which found that increasing *LFNG* dosage from knockdown to WT increases the rate of somitogenesis and the number of vertebrae [41].

In our proband, we counted over 20 vertebral bodies; this is more than what has been previously reported in others with loss of function *LFNG* variants. In these cases, the vertebral number appears to be less than 17 [29, 36]. In addition to a greater number of vertebral bodies, our proband is also taller (between 10^th^ and 25^th^ percentiles) than other reported cases (<5^th^ percentile) [24, 26, 27]. Parental heights for many of the cases are not reported. Finally, the arm span-to-height ratio in our proband was indicative of a more preserved trunk length (1.075) compared to previous reports (1.203) [26]. In the context of SCD3, arm span-to-height ratios closer to one indicate a lengthened trunk and thus a less pronounced phenotype. Together, these clinical features may highlight a partial rescue of trunk length, potentially due to the p.R174H substitution. However, the very small sample size and lack of certain molecular/phenotypic data prevent any definitive conclusions from being drawn. More evidence will need to be gathered to test whether vertebral number and trunk length can be recused by hypomorphic alleles in humans.

We did not observe a difference in vertebral body morphology compared to other reported cases with loss of function *LFNG* variants. Primarily, the “pebble beach sign,” a manifestation of rounded, offset vertebrae, was present (Figure 1(c)) (compare to [23–26]) [42]. A variety of segmentation defects, hemivertebrae, and rib abnormalities were also observed (Figure 1). Although we noted milder scoliosis in our proband, we cannot predict if this deformity will remain stable or progress with time (compare with [25–27]) [5, 43]. Therefore, with current evidence, it is unreasonable to suggest that segmentation defect severity is affected by the hypomorphic allele. Many more cases will need to be identified and fully molecularly characterized to test if segmentation defects, including scoliosis, are modulated by partially active LFNG enzyme.

### 4.4. Future Considerations in the Context of Variant Characterization

Our work highlights the importance of functional analyses for VUS alongside the difficulty in translating ambiguous functional results to the clinic. Here, we demonstrated that c.766G>A (p.G256S) causes a complete loss of LFNG function whereas c.521G>A (p.R174H) does not (Figure 3). Based on previous reports, homozygous c.766G>A variants would likely be pathogenic [24, 26, 27]; however, the pathogenicity of homozygous c.521G>A variants is much more difficult to predict. Our interpretation of the functional analysis, and that of many other genes, is limited by the current understanding of pathomechanisms [44]. Since the enzymatic activity threshold at which LFNG causes SCD3 is not known, the PS3 criteria are not entirely informative for c.521G>A. Therefore, we cannot discount the possibility of a milder SCD3 phenotype associated with this allele in a homozygous state. *LFNG* is not highly constrained (*Z* = 0.62, gnomAD 2.1.1), and thus, missense variants that lead to partial activity are worth further investigation. It would be scientifically and clinically informative to demarcate the threshold of LFNG activity required for proper somitogenesis.

Finally, although PS3 data can be required to inform probands and families of their reproductive risk status if they harbour VUS, these experiments are often costly, time-consuming, and technically difficult. In this study, this was largely due to creating, optimizing, and performing experiments with multiple epitope tags to ensure reliable and viable data. Therefore, we suggest that future research aims to identify high-throughput, reliable functional assays to limit lengthy and expensive variant characterization projects. One example of such an assay is saturation prime editing, a novel, high-throughput assay which can characterize hundreds to thousands of variants simultaneously [45]. Applying these novel techniques to genes which cause rare diseases could aid in limiting the cost and time commitment of variant characterization projects. Further, such projects would support the provision of informed consent during genetic counseling and reproductive risk assessment.

## 5. Conclusions

In this work, we were able to characterize two novel *LFNG* variants, c.521G>A (p.R174H) and c.766G>A (p.G256S). p.G256S is functionally null whereas p.R174H is functionally hypomorphic. To our knowledge, this is the first hypomorphic *LFNG* allele associated with human SCD3. Our evidence suggests that this hypomorphic variant may have partially rescued the proband's vertebral number and trunk length. We conclude that heightened LFNG activity may play a role in human SCD3 phenotypic variance. In the future, we hope to molecularly characterize all *LFNG* variants in the literature simultaneously to study the relationship between gene, protein, and pathological variance.

## Figures and Tables

**Table 1:** Novel *LFNG* variants associated with the SCD3 proband.

Variant	c.766G>A	c.521G>A
DNA change (GRCh38)	NC_000007.14:g.2525715G>A	NC_000007.14:g.2525258G>A
ClinVar ID	1003507	1062453
SNPdb ID	rs1437427476	N/A
LOVD DB ID	LFNG_000035	LFNG_000034
gnomAD v3.1.2	6.57 × 10^−6^	6.57 × 10^−6^
Protein	p.G256S	p.R174H
SIFT	Likely deleterious	Tolerated
PolyPhen-2	Damaging	Possibly damaging
Align-GVGD	Class C55	Class C25
CADD	25.6 (3.67^∗^)	25.6 (3.66^∗^)
REVEL	0.889	0.515

Note: *LFNG* transcript: RefSeq NM_001040167.2 (MANE select) (clone NP_001035257). ^∗^CADD raw score.

## Data Availability

The multiple sequence alignment data used to support the findings of this study are included within the supplementary information file. The genetic and phenotypic data used to support the findings of this study have been deposited in the LOVD repository (individual #00431334). The data were also submitted in ClinVar (accession numbers VCV001062453.5 and VCV001003507.5) by Invitae.

## Supplementary Materials

Supplementary Materials Supplementary Figure 1: ClustalX multiple sequence alignment of all clustered UniRef50 accessions related to LFNG. Displayed are 75 amino acids flanking the positions of interest labeled with a black column. The query sequence is the WT LFNG in both cases. (A) The MSA conserved residues surrounding position 256. Note the conservation of glycine throughout alignments. (B) The MSA conserved residues surrounding position 174. The bottom eight residues transition from LFNG sequences to MFNG sequences.
