# Supplementary material for: Functional Characterization of Novel Lunatic Fringe Variants in Spondylocostal Dysostosis Type-III with Scoliosis
Source: Hum Mutat. 2023 Jul 11;2023:5989733. doi: 10.1155/2023/5989733 (PMC11919168; doi:10.1155/2023/5989733)
Supplement: Supplementary Materials — Supplementary Figure 1: ClustalX multiple sequence alignment of all clustered UniRef50 accessions related to LFNG. Displayed are 75 amino acids flanking the positions of interest labeled with a black column. The query sequence is the WT LFNG in both cases. (A) The MSA conserved residues surrounding position 256. Note the conservation of glycine throughout alignments. (B) The MSA conserved residues surrounding position 174. The bottom eight residues transition from LFNG sequences to MFNG sequences. [file 5989733.f1.docx]

**Supplementary Figures**


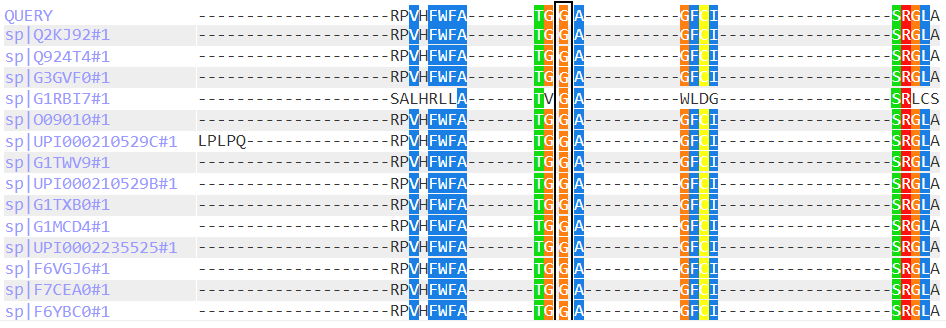
**A**

B


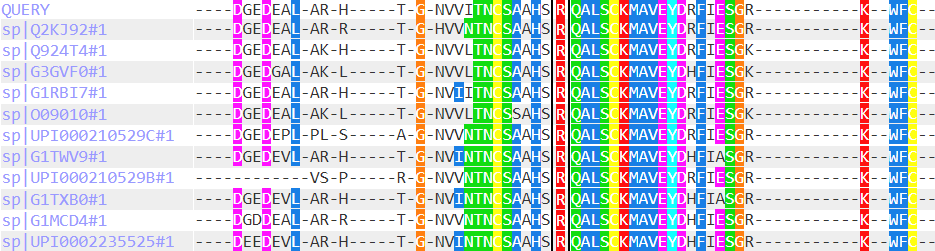


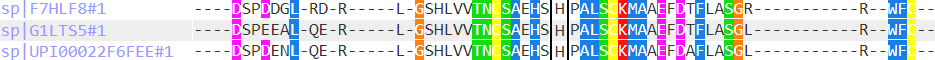


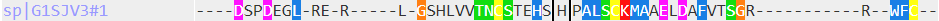


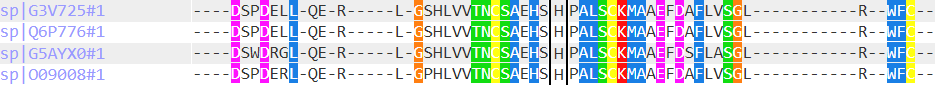


**Supplementary Figure 1: ClustalX multiple sequence alignment of all clustered UniRef50 accessions related to LFNG**.
